# Peptides derived from *Plasmodium falciparum* leucine-rich repeat 1 bind to serine/threonine phosphatase type 1 and inhibit parasite growth in vitro

**DOI:** 10.2147/DDDT.S153095

**Published:** 2018-01-09

**Authors:** Christine Pierrot, Xiguang Zhang, Gigliola Zhangi, Aline Fréville, Angelita Rebollo, Jamal Khalife

**Affiliations:** 1 Center for Infection and Immunity of Lille, U1019 – UMR 8204, Institut Pasteur de Lille, Université de Lille, Lille Cedex; 2 CIMI Paris, UPMC/Inserm U1135, Paris, France

## Introduction

The biogenesis of protein phosphatase 1 (PP1) holoenzyme in eukaryotes requires diverse regulatory subunit proteins (RSPs) that bind to the highly conserved PP1 catalytic subunit (PP1c) and direct its spatiotemporal activity as well as its specificity. Several studies demonstrated that most RSPs share a canonical common binding motif, the RVXF motif, which is present in ~85% of RSPs and is considered as the main contributor for the interaction to PP1c.^1^ In *Plasmodium falciparum* (Pf), our earlier studies revealed that leucine-rich repeat 1 (LRR1), one of the major RSPs of PfPP1 and an ortholog of human and yeast Sds22, lacks the RVXF motif. The amino acids sequence of PfLRR1 exhibits nine leucine-rich repeats (LRRs) and a hydrophobic region at the C-terminal end, known as the LRR cap motif.^2^ In this work, we identified the PP1-binding peptides of PfLRR1 and examined their capacity to affect Pf growth.

## Methods

### Peptides

Peptides were synthesized in an automated multiple peptide synthesizer with solid-phase procedure and standard fluorenylmethyloxycarbonyl chemistry. The purity and composition of the peptides were confirmed by reverse-phase high performance liquid chromatography and by amino acid analysis.

### PP1-binding assays on cellulose-bound peptides containing LRR1 sequence

Overlapping dodecapeptides scanning the whole LRR1 sequence were prepared by automated spot synthesis (Abimed, Langerfeld, Germany) onto an amino-derived cellulose membrane, as described.^3^,^4^ The membrane was developed as described by Tian et al.^5^

### Binding of PfPP1 with synthetic-derived peptides from PfLRR1 using an enzyme-linked immunosorbent based-assay

The method has been described previously in detail and was used without any modification.^6^,^7^

### Parasite culture and growth inhibition assay of blood-stage parasites

These methods have been extensively described.^6^

### Pf sporozoites

Pf (NF54) sporozoites were isolated by aseptic dissection of the salivary glands of infected *Anopheles stephensi* obtained from the Department of Medical Microbiology, University Medical Centre, St Radboud, Nijmegen, the Netherlands.

### Primary hepatocyte in vitro cultures

Human primary hepatocytes were isolated from liver segments obtained from adult patients undergoing partial hepatectomy as described by Dembélé et al.^8^

### Infection and drug assays

The protocol has been described by Barata et al.^9^

### Parasite quantification

Pre-erythrocytic parasites were detected by immunofluorescence following the protocol described by Dembélé et al.^8^

## Results and discussion

**Figure 1:**
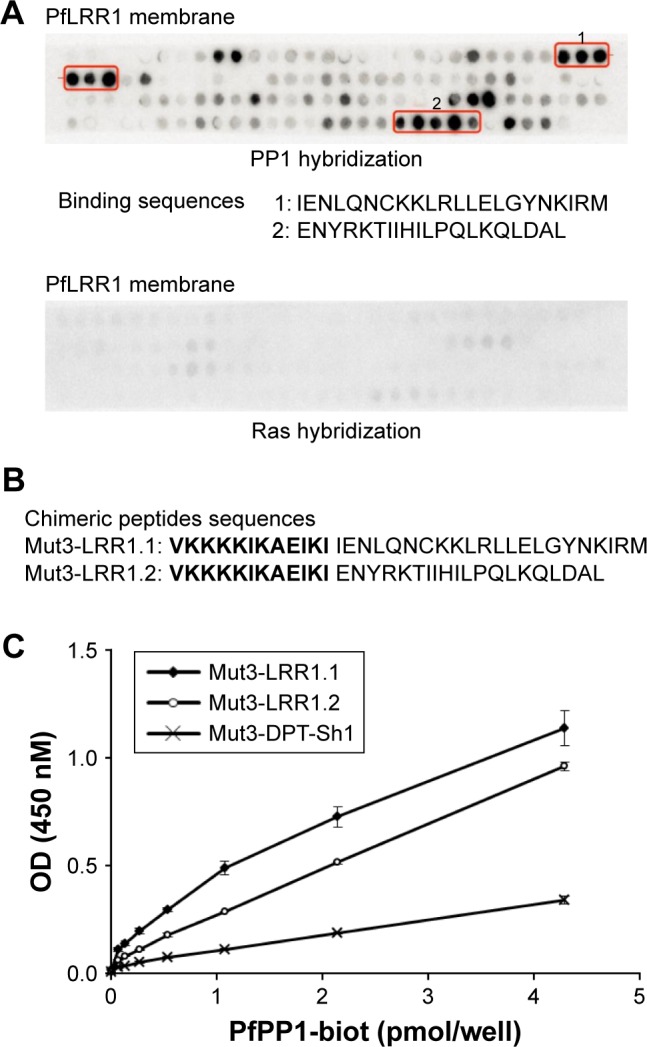
(**A**) Mapping of PfLRR1–PP1 interaction. Overlapping dodecapeptides with two amino acids shift covering PfLRR1 sequence were bound to a solid support. The membrane was incubated sequentially with PP1 protein and anti-PP1 antibody, followed by a secondary antibody. The membrane was revealed using the ECL system. As a control, the membrane was also hybridized with the irrelevant protein Ras. (**B**) Sequences of chimeric penetrating peptides (penetrating sequence is bolded). (**C**) Binding of chimeric peptides to PfPP1 analyzed in an ELISA-based assay. Peptides were coated in 96-well plates and incubated with biotinylated recombinant PfPP1. Results are from one representative experiment out of two (mean ± standard deviation). LRR1.1 indicates site 1; LRR1.2 indicates site 2. **Abbreviations:** Pf, *Plasmodium falciparum*; LRR1, leucine-rich repeat 1; PP1, protein phosphatase 1; PfPP1-biot, biotinylated PfPP1; ECL, enhanced chemiluminescence; ELISA, enzyme-linked immunosorbent assay.

To better define the PP1-binding sites of PfLRR1, we performed a peptide array screening with overlapping dodecapeptides, including the LRR cap. Dot blot analysis presented in Figure 1A revealed that two sites were able to bind to PP1. The first PP1c-binding sequence of PfLRR1, IENLQNCKKLRLLELGYNKIRM, contains an LRR motif, and the second sequence, ENYRKTIIHILPQLKQLDAL, corresponds to the LRR cap of the protein. The specificity of this binding was confirmed by the absence of binding of an irrelevant protein (Figure 1A).

Based on these results, the ability of the two binding peptides to inhibit Pf growth was then examined. To this end, we synthesized peptides containing the shuttle sequence VKKKKIKAEIKI (Mut3-DPT-Sh1), known to deliver peptides to cells,^10^ and the binding sequence 1 (Mut3-LRR1.1) or 2 (Mut3-LRR1.2) (Figure 1B). To evaluate whether these chimeric cell-penetrating and cell-interfering peptides retained their capacity to bind to PP1, their binding to recombinant PfPP1 was first explored. Results presented in Figure 1C confirmed that PfPP1 was able to bind to Mut3-LRR1.1 and Mut3-LRR1.2. These results differ from those of human Sds22 structure-binding studies. Indeed, single-point mutations in LRRs of human Sds22 and synthetic peptides showed that the PP1-binding regions extended over the last six LRRs.^11^,^12^ In the case of PfLRR1, our data showed for the first time that, besides the engagement of one LRR motif, the LRR cap seems to contribute to the interaction of PfLRR1 with PP1.

**Figure 2:**
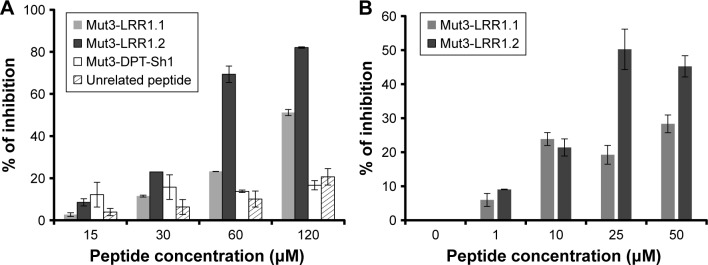
In vitro effect of PfLRR1-derived peptides on Pf growth. (**A**) In vitro effect of chimeric peptides on the erythrocytic stages of Pf. Peptides at different concentrations were incubated with Pf3D7-infected erythrocytes for 48 h. Two control peptides were used: Mut3-DPT-Sh1 (VKKKKIKAEIKI) and an unrelated peptide from PF3D7_1010600 gene (VKKKKIKREIKIFGEKKKFGEKKK). Parasitemia was measured by flow cytometry analysis after SYBR Green I staining. Controls for the calculation of % of inhibition were obtained with 500 nM chloroquine (100% of inhibition) or medium (0% of inhibition). Results of one representative experiment out of three are shown as mean ± standard deviation. (**B**) In vitro effect of chimeric peptides on hepatic stages of Pf. Sporozoites were obtained from infected salivary glands of *Anopheles stephensi*. Human hepatocytes were isolated from liver segments obtained from adult patients undergoing partial hepatectomy. Different concentrations of peptides were added to the culture medium 3 h after infection of hepatocytes with Pf sporozoites and renewed every day for 5 days. Following fixation, the culture plates were then stained with anti-Pf hsp70 polyclonal antibody followed by a fluorochrome-labeled secondary antibody. Parasites were detected under fluorescence microscope. Results of one representative experiment out of two are shown as mean ± standard deviation. LRR1.1 indicates site 1; LRR1.2 indicates site 2. **Abbreviations:** Pf, *Plasmodium falciparum*; LRR1, leucine-rich repeat 1.

Next, we examined the effect of these chimeric peptides on the growth of Pf in vitro. As shown in Figure 2A, the peptide Mut3-LRR1.2 inhibited around ~80% the growth of Pf blood-stage parasites at the highest concentration. The peptide Mut3-LRR1.1 exhibited an inhibition of ~50%. The control Mut3-DPT-Sh1 showed only an inhibition of ~10% at any concentration used.

The antiparasitic activity of the two chimeric peptides was also tested on the in vitro hepatic stage of Pf. Figure 2B shows that both peptides have a significant inhibitory activity on Pf liver-stage development, but that the Mut3-LRR1.2 peptide is more active, as in the case for the blood stage. These data could be explained by the capacity of binding motifs to disrupt/destabilize PfLRR1–PfPP1 interaction in Pf, leading to parasite growth inhibition.

The evaluation of the toxicity of both peptides analyzed on primary human hepatocytes at different concentrations that were active on parasites showed that they did not affect the host cell viability (data not shown). Taken together, these results confirm that these two peptides have specific antiplasmodial activity on both Pf erythrocytic and hepatic stages. In addition, these observations support our previous data showing the antiplasmodial capacity of RVXF-derived peptides^6^ and raise the possibility for the synthesis of stable, administrable peptides affording high selectivity/potency and/or small molecules specifically targeting Pf RSPs of PP1c.
