# Peptides derived from *Plasmodium falciparum* leucine-rich repeat 1 bind to serine/threonine phosphatase type 1 and inhibit parasite growth in vitro [Corrigendum]

**DOI:** 10.2147/DDDT.S165760

**Published:** 2018-03-29

Pierrot C, Zhang X, Zhangi G, Fréville A, Rebollo A, Khalife J. Drug Des Devel Ther. 2018;12:85–88.

On page 1, the author’s name was incorrectly listed as Gigliola Zhangi. Their correct name is Gigliola Zanghi.

